# Leaf-Wounding Long-Distance Signaling Targets AtCuAOβ Leading to Root Phenotypic Plasticity

**DOI:** 10.3390/plants9020249

**Published:** 2020-02-15

**Authors:** Ilaria Fraudentali, Renato A. Rodrigues-Pousada, Paraskevi Tavladoraki, Riccardo Angelini, Alessandra Cona

**Affiliations:** 1 Department of Science, University “Roma Tre”, 00146 Rome, Italy; ilaria.fraudentali@uniroma3.it (I.F.); paraskevi.tavladoraki@uniroma3.it (P.T.); riccardo.angelini@uniroma3.it (R.A.); 2 Department of Life, Health and Environmental Sciences, University of L’Aquila, 67100 L’Aquila, Italy; pousada@univaq.it

**Keywords:** Copper amine oxidases, polyamines, hydrogen peroxide, wounding, root plasticity, protoxylem

## Abstract

The Arabidopsis gene *AtCuAOβ* (At4g14940) encodes an apoplastic copper amine oxidase (CuAO) highly expressed in guard cells of leaves and flowers and in root vascular tissues, especially in protoxylem and metaxylem precursors, where its expression is strongly induced by the wound signal methyl jasmonate (MeJA). The hydrogen peroxide (H_2_O_2_) derived by the AtCuAOβ-driven oxidation of the substrate putrescine (Put), mediates the MeJA–induced early root protoxylem differentiation. Considering that early root protoxylem maturation was also induced by both exogenous Put and leaf wounding through a signaling pathway involving H_2_O_2_, in the present study we investigated the role of *AtCuAOβ* in the leaf wounding-induced early protoxylem differentiation in combination with Put treatment. Quantitative and tissue specific analysis of *AtCuAOβ* gene expression by RT-qPCR and promoter::*green fluorescent protein-β-glucuronidase* fusion analysis revealed that wounding of the cotiledonary leaf induced *AtCuAOβ* gene expression which was particularly evident in root vascular tissues. *AtCuAOβ* loss-of-function mutants were unresponsive to the injury, not showing altered phenotype upon wounding in comparison to wild type seedlings. Exogenous Put and wounding did not show synergy in inducing early root protoxylem maturation, suggesting their involvement in a shared signaling pathway.

## 1. Introduction

Efficient plant resistance to environmental stresses relies on rapid stimulus perception and transmission within and among cells. Bidirectional extensive root-shoot communication allows plants to respond as whole organisms, coordinating and integrating reversible rapid physiological responses to phenotypic plasticity expression in different tissues. Wounding-induced long-distance signaling ensures defense responses throughout the plant body, preparing distal tissues for arriving chewing herbivores. Upon herbivore attack, infested plants undergo changes in primary and secondary metabolism, and a variety of defense compounds increase in concentration both in leaves and roots, with belowground organisms inducing defense responses aboveground and vice versa [1,2]. The rapid systemic accumulation of the wound/herbivore signal jasmonic acid (JA) is triggered by leaf-to-leaf [3,4,5] or root-to-shoot [6] long-distance signaling propagated by electrical and/or reactive oxygen species waves [5,6]. In roots, the wound-induced burst of JA occurs at a much lower extent than in leaf [7,8], possibly due to the lower level of α-linoleic acid in root plastid membranes [9], and JA accumulation mainly depends on locally [1] or systemically-induced [6] JA biosynthesis in shoots. Noteworthy, root resistance against the nematode *Meloidogyne incognita,* relies on a root-shoot-root communication loop by which root-to-shoot electric waves generated in infested roots trigger biosynthesis of JA in leaves, which moves back to roots leading to JA accumulation and enhanced resistance against the rhizosphere herbivore [6].

Among systemic long-term responses to leaf wounding, the occurrence of root protoxylem phenotypic plasticity [10] has been recently reported in Arabidopsis (*Arabidopsis thaliana*). The early root protoxylem differentiation described after leaf wounding, which was highlighted as changes in protoxylem position that appeared closer to the root tip [10], was consistent with the early root protoxylem differentiation observed after treatment with methyl jasmonate (MeJA) [11]. Plasticity in root growth and architecture may be functional under water-limited environmental conditions [12], enhancing water absorption from the soil [13]. As expression of many wound-inducible genes is induced by dehydration [14] and JA level increases in tissues undergoing water stress [15], it is possible that leaf wounding activates dehydration signaling in roots leading to the expression of xylem phenotypic plasticity.

The early root protoxylem differentiation occurring after both leaf wounding and MeJA treatment was shown to be signaled by hydrogen peroxide (H_2_O_2_) that in the case of the MeJA-induced event derives from the Arabidopsis copper amine oxidase β (AtCuAOβ)-mediated oxidation of its preferential substrate, the polyamine (PA) putrescine (Put) [11,16]. Involvement of amine oxidases (AOs)-driven production of H_2_O_2_ in early xylem differentiation was observed also in maize (*Zea mays*) and tobacco (*Nicotiana tabacum*) roots under stress-simulated conditions, such as those induced by AO-overexpression or PA-treatment [17], as well as those signaled by a compromised status of cell-wall pectin integrity [18]. Final differentiation of xylem vessel precursors culminates with programmed cell death events that require an accurate spatio-temporal coordination with cell wall lignification events to produce functional vessels, with both these two terminal key events depending on developmental- and/or stress-driven H_2_O_2_ production. Here, we provide evidence that the AtCuAOβ-driven H_2_O_2_ production mediates the early root protoxylem differentiation signaled by shoot-to-root long-distance communication upon leaf wounding.

## 2. Results

### 2.1. Leaf Wounding Induces AtCuAOβ Expression in the Root

**Figure 1:**
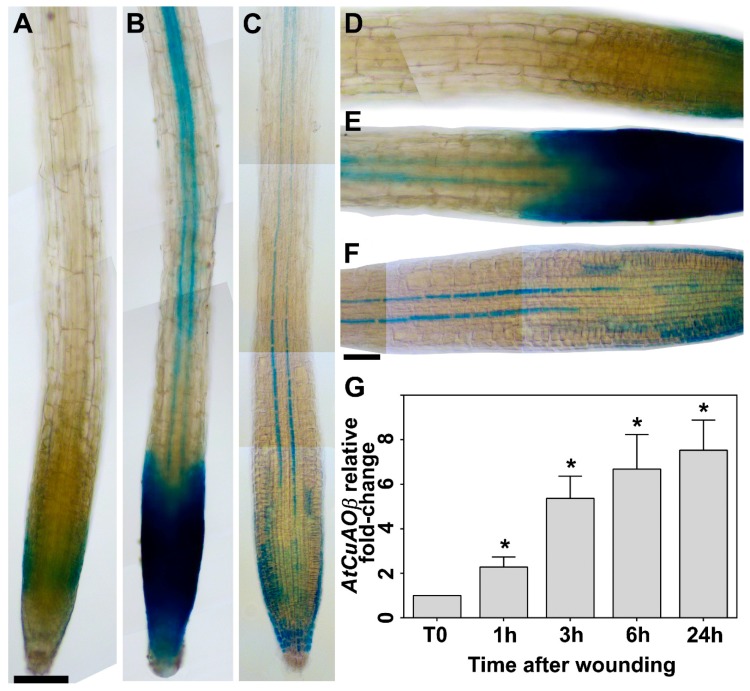
Analysis of *AtCuAOβ* gene expression upon leaf wounding by GUS staining analysis (root) and RT-qPCR (whole seedlings). (**a**–**f**) Light microscopy analysis after GUS staining of roots from 7-day-old *AtCuAOβ-promoter::GFP-GUS* seedlings unwounded (**a**–**f**) or leaf-wounded after 3 h from the injury (**b**,**e**). The staining reaction was allowed to proceed for 2 h (**a**,**d** unwounded, and **b**,**e** wounded leaf) or overnight (**c**,**f**; unwounded). Shown images were obtained aligning serial overlapping micrographs of the same root using Photoshop Software (Adobe). Ten plants from three independent experiments were analyzed and images from a single representative experiment is shown. Bar: 200 μm (**a**–**c**); 100 μm (**d**–**f**). (**g**) RT-qPCR analysis in 7-day-old wild type (WT) whole seedlings at 0, 1, 3, 6 and 24 h time-points from cotyledonary leaf injury. Three biological replicates each with three technical replicates were performed (*n* = 3). *AtCuAOβ* mRNA level after wounding is relative to that of the corresponding unwounded plant for each time point. The significance levels (*p*-values) between the relative mRNA level at each time and the mRNA level of control unwounded plant at time 0 (T0), which is assumed to be one, have been calculated with one-way analysis of variance (ANOVA) followed by Sidak’s multiple comparison test (*; *p*-values ≤ 0.05).

Considering that *AtCuAOβ* expression is induced by the wound-signal hormone MeJA, especially in the root vascular tissues [11], here, *AtCuAOβ* gene expression profile upon cotyledonary leaf wounding was investigated by *AtCuAOβ-promoter::GFP-GUS* transgenic analysis and reverse transcription-quantitative polymerase chain reaction (RT-qPCR) (Figure 1). Tissue specific expression analysis of *AtCuAOβ-promoter::GFP-GUS* plants wounded on cotyledonary leaf showed a strong induction in the vascular tissues at the transition, elongation and maturation root zone, as well as in root cap (Figure 1b,e), as compared to control unwounded plants (Figure 1a,d). The tissue-specific pattern revealed after wounding was similar to that observed in unwounded seedlings after prolonged staining (24 h) (Figure 1c,f), showing a similar pattern to that previously revealed in 4-day-old plants [11]. Consistently, RT-qPCR analysis shows a strong progressive induction of *AtCuAOβ* gene expression levels from 1 to 24 h, with a 2-, 5-, 6.5- and 7.5-fold increase, respectively, compared to T0 levels (Figure 1g).

### 2.2. The H_2_O_2_-Mediated Early Root Protoxylem Maturation Upon Leaf Injury is Impaired in AtCuAOβ Mutants

**Figure 2:**
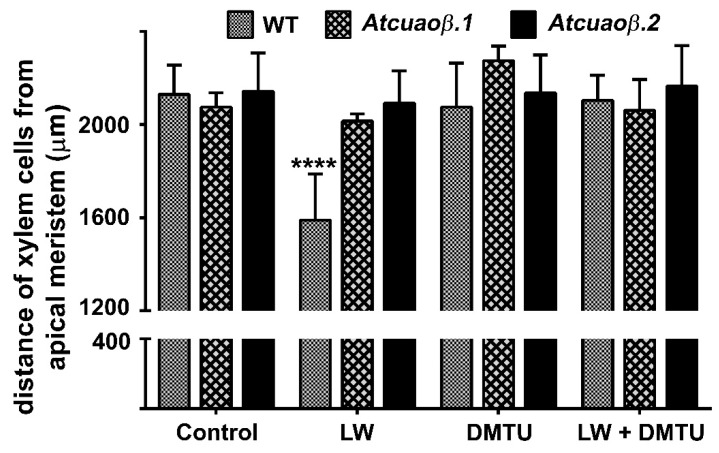
Analysis of differences in protoxylem maturation in 10-day-old leaf-wounded (LW) WT and mutant (*Atcuaoβ.1* and *Atcuaoβ.2*) seedlings, grown in medium with or without the H_2_O_2_-scavenger DMTU at a final concentration of 100 μM. Distances from the apical meristem of the protoxylem position (defined by the position of the first protoxylem cell with fully developed secondary cell wall thickenings) are reported. These experiments were repeated at least three times with 10 seedlings analyzed each time (mean values ± SD; *n* = 30). The statistical significance levels (*p*-values) were evaluated with one-way analysis of variance (ANOVA) followed by Sidak’s multiple comparison test (****, *p* ≤ 0.0001). Insignificant differences are not indicated.

To explore the role played by *AtCuAOβ* in leaf wounding-triggered alteration of root phenotypic plasticity, 7-day-old Arabidopsis wild type (WT) and insertional *Atcuaoβ* mutant seedlings were treated or not with the H_2_O_2_-scavenger *N,N^1^*-dimethylthiourea (DMTU) and then injured by cutting a cotyledonary leaf. After 3 days from injury, roots were observed under Laser Scanning Confocal Microscope (LSCM) to investigate protoxylem position and meristem size after staining with propidium iodide (PI) that highlight cell wall. Figure 2 shows that, in physiological conditions, roots of both *Atcuaoβ* mutants present no apparent altered phenotype in xylem tissues compared to WT roots, in accordance with what has been previously reported [11]. Cotyledonary leaf wounding induced early protoxylem differentiation in roots of WT plants, consistently with data previously shown [10], while not affecting protoxylem differentiation in both *Atcuaoβ* mutants (Figure 2). In fact, the mean distance of the first protoxylem cell with fully developed secondary wall thickenings from the root apical meristem (hereafter referred as protoxylem position [10,11]) was approximately 1600 µm in WT leaf-wounded plants as compared to unwounded control WT and mutant plants (Figure 2 and [10]) and wounded mutant plants showing a distance of approximately 2000/2100 µm (Figure 2).

Moreover, DMTU treatment while reversing the leaf wounding-induced early protoxylem differentiation in WT plants (Figure 2 and [10]) had no effect in wounded-*Atcuaoβ* mutants (Figure 2). Treatment with DMTU alone did not affect the protoxylem differentiation of either WT or *Atcuaoβ* mutants (Figure 2 and [10]). Confirming previous data showing leaf wounding ineffectiveness in altering root growth and meristem size in WT plants [10], both parameters (Supplementary Materials Tables S1 and S2) were unchanged in leaf-wounded/DMTU-treated mutant plants as compared to unwounded/untreated control mutant plants.

**Figure 3:**
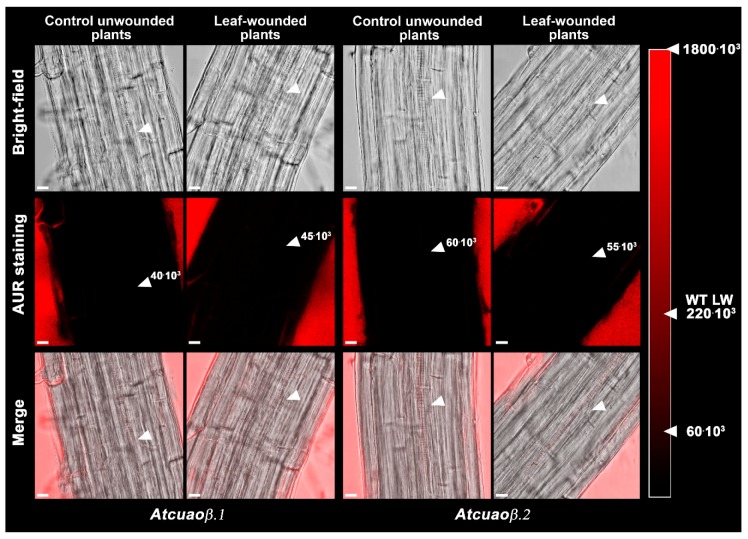
In situ H_2_O_2_ detection by LSCM analysis, after AUR staining, of roots from 7-day-old unwounded control and leaf-wounded mutant seedlings (*Atcuaoβ.1* and *Atcuaoβ.2*) 6 h after the injury. The corresponding bright-field and overlay images are shown. Micrographs show the root zone corresponding to the site of appearance of the first protoxylem cell with fully developed secondary cell wall thickenings (arrows) and have been taken at the level of the central root section. Images are representative of those obtained from ten seedlings from three independent experiments. The average values of fluorescence intensity measured as the sum of the pixels of each of five 65 μm^2^ rectangle are reported for each condition (mean values ± SD; *n* = 25). The maximum pixel sum for a completely saturated square was approximately 1800 × 10^3^. In the red degrading scale are reported for comparison the average values of fluorescence intensity for unwounded control and leaf-wounded (LW) WT plants that respectively were 60 × 10^3^ ± 19 × 10^3^ and 220 × 10^3^ ± 38 × 10^3^ (data from [10]). Bars: 10 μm.

Furthermore, to verify the involvement of H_2_O_2_ produced via *AtCuAOβ*-driven PA oxidation in the wound-induced-signaling pathways leading to early root protoxylem differentiation, H_2_O_2_-dependent Amplex Ultra Red (AUR) fluorescence assay was performed in 7-day-old roots of *Atcuaoβ* mutants wounded or not on the cotyledonary leaf (Figure 3). Neither in roots of unwounded nor in those of leaf-wounded *Atcuaoβ* mutants, H_2_O_2_-dependent AUR fluorescence was detectable at the site of differentiating protoxylem elements (Figure 3; Supplementary Materials Figure S1), as compared to roots of WT wounded plants where clear AUR staining was detectable [10].

### 2.3. Exogeneous Put and Wounding do Not Act Synergistically in inducing Early Root Protoxylem Maturation

**Figure 4:**
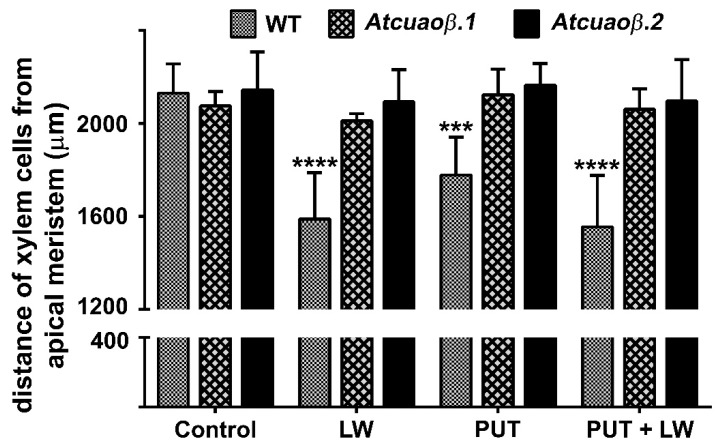
Analysis of differences in protoxylem maturation in 10-day-old leaf-wounded (LW) WT and mutant (*Atcuaoβ.1* and *Atcuaoβ.2*) seedlings, grown in medium with or without 100 µM Put. Distances from the apical meristem of the protoxylem position (defined by the position of the first protoxylem cell with fully developed secondary cell wall thickenings) are reported. These experiments were repeated at least three times with ten seedlings analyzed each time (mean values ± SD; *n* = 30). The statistical significance levels (*p*-values) were evaluated with one-way analysis of variance (ANOVA) followed by Sidak’s multiple comparison test (****, *p* ≤ 0.0001; ***, *p* ≤ 0.001). Insignificant differences are not indicated.

In order to obtain further evidence of the involvement of AtCuAOβ activity in xylem differentiation, the AtCuAOβ substrate Put [19] was provided at the concentration of 100 μM to unwounded and wounded WT and *Atcuaoβ* mutant seedlings. After 3 days from the injury by cutting a cotyledonary leaf, roots were observed under LSCM, for the investigation of the protoxylem position and meristem size. Figure 4 shows that treatment with 100 μM Put induced early protoxylem differentiation in WT plants (protoxylem position at 1800 μm from the apical meristem) coherently with what previously reported [11] but not in *Atcuaoβ* mutants as compared with the corresponding untreated plants (Figure 4). No additive/synergic effect in respect to either leaf-wounded or Put-treated plants was observed (Figure 4). Indeed, combination between Put and cotyledonary leaf-wounding in WT seedlings did not alter protoxylem position, which was identified at similar distance to the wounded-only seedlings (protoxylem position at 1600 μm from the apical meristem) (Figure 4). This would suggest that a plateau effect on protoxylem position has been reached upon leaf wounding. Instead, protoxylem position in *Atcuaoβ* mutants was not altered by Put, either alone or combined with wounding.

Confirming previous data showing Put treatment ineffectiveness in altering root growth and meristem size in WT plants [11], both parameters (Supplementary Materials Tables S1 and S2) were unchanged in leaf-wounded/Put-treated mutant plants as compared to unwounded/untreated control mutant plants.

**Figure 5:**
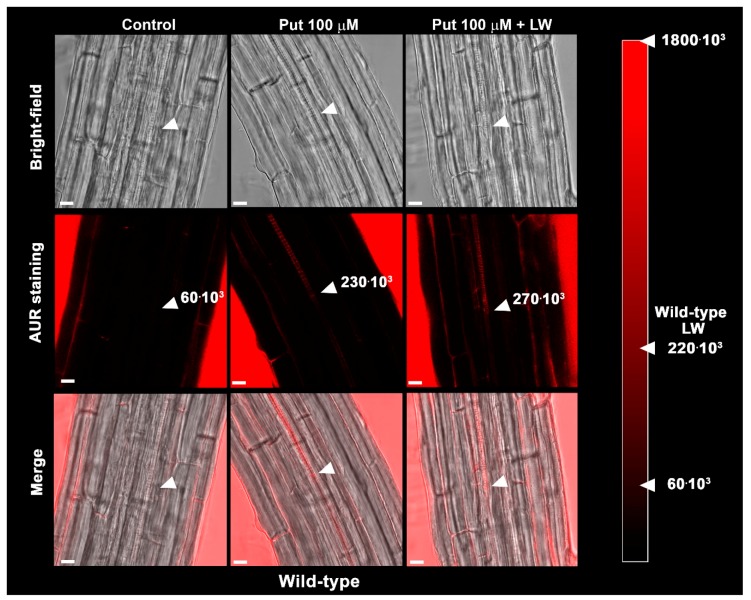
In situ H_2_O_2_ detection by LSCM analysis, after AUR staining, of roots from 7-day-old unwounded Put-untreated control (Control), unwounded Put-treated (Put 100 µM) and leaf-wounded Put-treated (Put 100 µM + LW) WT seedlings. Treatment with 100 µM Put was performed 6 h after the injury. The corresponding bright-field and overlay images are shown. Micrographs show the root zone corresponding to the site of appearance of the first protoxylem cell with fully developed secondary cell wall thickenings (arrows) and have been taken at the level of the central root section. Images are representative of those obtained from ten seedlings from three independent experiments. The average values of fluorescence intensity measured as the sum of the pixels of each of five 65 μm^2^ rectangle are reported for each condition (mean values ± SD; *n* = 25). The maximum pixel sum for a completely saturated square was approximately 1800 × 10^3^. In the red degrading scale are reported for comparison the average values of fluorescence intensity for leaf-wounded (Put-untreated) WT plants, respectively, were 60 × 10^3^ ± 19 × 10^3^ and 220 × 10^3^ ± 38 × 10^3^ (data from [10]). Bars: 10 μm.

Figure 5 shows that 6 h after treatment with 100 µM Put, a strong AUR signal was revealed in the root zone where the first protoxylem cell with fully developed secondary cell wall thickenings is found in WT, which was not detectable in roots of unwounded Put-untreated control WT and unwounded and wounded Put-treated *Atcuaoβ* mutant seedlings (Figure 5 and Supplementary Materials Figure S2). Moreover, the combination of cotyledonary leaf wounding and Put treatment in WT seedlings did not seem to increase H_2_O_2_ accumulation at the site of differentiating protoxylem elements compared to wounding or Put treatment alone, suggesting that the plateau effect reached in terms of protoxylem position is correlated to the H_2_O_2_ production induced by leaf wounding (Figure 5, Supplementary Materials Figures S1 and S2).

## 3. Discussion

Present results provide the evidence that AtCuAOβ is the source of the apoplastic H_2_O_2_ implicated in the leaf wounding-induced early root protoxylem-differentiation, signaled by shoot-to-root long-distance communication. AtCuAOβ is a cell wall resident AO expressed in guard cells and root xylem tissues [11,20] where it is involved in the terminal PAs catabolism. PAs undergo oxidative deamination by AOs, which are represented by a large enzymatic class including not only CuAOs but also flavin adenine dinucleotide (FAD)-dependent polyamine oxidases (PAOs) [21]. Heterogeneity in molecular properties, subcellular localization and tissue specific expression of each AO member/family reflects in a multitude of physiological roles accomplished by shared strategies of action via both control of polyamine homeostasis and delivery of biologically-active compounds, among which H_2_O_2_ [21,22,23].

In the cell wall, the absence/low levels of free available PAs [24] suggests a prevalent role of AOs in H_2_O_2_ delivering, depending on developmentally-regulated or stress-induced PA secretion into the apoplast and/or AO redistribution from cytoplasm towards cell wall [25,26,27,28,29,30,31]. Apoplastic PA-derived H_2_O_2_ play a dual role in triggering peroxidase-mediated wall stiffening events [32] and signaling modulation of gene expression [33], especially regarding defense and hypersensitive response (HR)-cell death genes [34,35]. In xylem tissue, stress-induced PA-derived H_2_O_2_ could trigger developmental cell death and act as co-substrate in peroxidase-mediated lignin polymerization, prompting final differentiation of xylem vessel precursors.

In line with what previously reported for maize (*Zea mays*) PAO [36] and chickpea (*Cicer arietinum*) CuAO [37] mRNA, protein and enzyme activity levels after wounding, a strong induction of *AtCuAOβ* gene expression upon leaf wounding was revealed by RT-qPCR analysis (Figure 1g). Furthermore, the strong induction of *AtCuAOβ* gene expression highlighted by GUS staining in the root of leaf-wounded *AtCuAOβ-promoter::GFP-GUS* plants (Figure 1b,e), suggests a role of this gene as target of the long-distance leaf-to-root signaling leading to root phenotypic plasticity. In detail, the tissue-specific profile of *AtCuAOβ* expression revealed a strong staining in the vascular tissues at the transition, elongation and maturation root zone that resembles the profile of the MeJA-induced *AtCuAOβ* gene expression [11], and strongly supports the hypothesis of an *AtCuAOβ* role in leaf wounding-induced early root protoxylem differentiation. A possible role of *AtCuAOβ* in lignification events occurring in developing tracheary elements was firstly suggested on the basis of a histochemical analysis showing overlapping profiles between *AtCuAOβ* expression and lignification pattern [19], while the role in developmental programmed cell death (PCD) was suggested by the high expression levels found in cells fated to undergo PCD, such as developing vascular elements and lateral root cap cells that are continually displaced and sloughed off [19]. The involvement of AOs in developmental PCD was also supported by the evidence that spermidine-derived H_2_O_2_ was shown to induce nuclear condensation and DNA fragmentation in maize primary roots by the means of terminal deoxynucleotidyl transferase dUTP nick end labeling (TUNEL) assay [17] and by the high PAO protein and enzyme activity levels found in maize root cap [31]. Noteworthy, the intense GUS staining detected in Arabidopsis root cap after leaf wounding (Figure 1b,e) may reveal the need for an increased *AtCuAOβ*-mediated H_2_O_2_ production due to an induction of PCD events in lateral root cap cells by leaf wounding, similarly to what described for other environmental stresses including salt stress and drought [38,39].

The analysis of protoxylem position revealed that *AtCuAOβ* mutants are unresponsive to the leaf-wounding induction of early protoxylem differentiation in roots, as protoxylem position did not change in both mutants upon injury (Figure 2). The absence of H_2_O_2_-dependent AUR fluorescence at the site of differentiating protoxylem elements in roots of leaf-wounded *AtCuAOβ* mutants supports the central role of the *AtCuAOβ*-driven H_2_O_2_ production in the leaf wounding-induced early root protoxylem differentiation as alternative pathway in terminal differentiation of xylem vessel precursors under stress. Otherwise, the absence of H_2_O_2_-dependent AUR fluorescence in control unwounded WT and *AtCuAOβ* mutants ([10]; Figure 3) confirm the occurrence of a developmentally-controlled H_2_O_2_-independent signaling pathway governed by the auxin/cytokinin/T-Spm loop [40,41] under physiological conditions. Further evidence supporting the hypothesis of alternative/integrative signaling pathways leading to terminal differentiation of xylem vessel precursors under physiological or stress conditions is provided by the evidence that the H_2_O_2_ scavenger DMTU, while reversing the leaf-wounding induced root protoxylem differentiation in WT plants, is ineffective in inducing any variation in root protoxylem position both in control unwounded WT and mutant plants ([10]; Figure 2), when the physiological loop is possibly operative. It is also ineffective in leaf-wounded mutant plants (Figure 2) in which the absence of *AtCuAOβ*-driven H_2_O_2_ production hinder the stress-induced early protoxylem differentiation. Root growth and meristem size were unchanged after wounding or DMTU treatment either alone or in combination (Supplementary Materials Tables S1 and S2), similarly to what was previously demonstrated for WT plants [10], confirming that this H_2_O_2_-independent signaling pathway is uncorrelated with the H_2_O_2_-dependent pathway effective in modulating protoxylem position under stress. In line with previous results carried out on 14-day-old WT and mutant seedlings [11], treatment with Put was effective in moving protoxylem position towards the root tip (Figure 4), even though at a lesser extent with respect to leaf wounding (Figure 4), concurrently inducing a strong AUR signal in the root zone where the first protoxylem cell with fully developed secondary cell wall thickenings is found (Figure 5) in WT plants while being ineffective in mutants (Supplementary Materials Figure S2). However, Put supply and leaf-wounding in combined treatment did not exert any additive/synergic effect in respect to leaf-wounded plants, suggesting that Put is a likely component of the signaling pathway triggered by leaf wounding, which alone exerted a saturating effect on root protoxylem differentiation. Indeed, a synergic effect would be expected in the case of two independent Put-induced/wound-induced signaling pathways. In support of this, the AUR fluorescence-revealed H_2_O_2_ accumulation (Figure 5) did not increase following the combined treatment in correlation with protoxylem position. This data would support Put as the PA involved in the AtCuAOβ driven-H_2_O_2_ production. Overall, the above-reported results involve the H_2_O_2_ produced by the AtCuAOβ-mediated Put oxidation in the long-distance signaling pathway linking a distal abiotic stress such as leaf wounding to root protoxylem phenotypic plasticity and open the question of unravelling the physiological meaning of this alteration. Indeed, although some hypotheses have been reported putting in relation root growth and xylem plasticity to water uptake efficiency in improving drought tolerance [13], the physiological role of variation in protoxylem position after wounding deserves to be further analyzed.

## 4. Materials and Methods

### 4.1. Plant Materials, Treatments and Root Growth Analysis

The Columbia-0 (Col-0) ecotype of Arabidopsis (*Arabidopsis thaliana*) was used as the wild type (WT). The Arabidopsis Col-0 T-DNA insertion lines *Atcuaoβ.1* (SALK_145639.55.25.x; TAIR accession no. 1005841762; previously *Atao1.1* [11]) and *Atcuaoβ.2* (SALK_077391.40.85.x; TAIR accession no. 4284859; previously *Atao1.2* [11]) of the *CuAO* gene At4g14940 (*AtCuAOβ*; TAIR accession no. 2129519; [21]) that were used were obtained and characterized as previously described [11].

Plants were grown in vitro in a growth chamber at a temperature of 23 °C under long-day conditions (16/8 h photoperiod; 50 μmol m^−2^ s^−1^ and 55% relative humidity). For in vitro growth, seeds were surface sterilized as previously described [42]. After extensive washing with sterile water, seeds were stratified at 4 °C for 2 days in the dark and then sown in ½ Murashige and Skoog (MS) salt mixture (pH 5.7) supplemented with 0.5 (w/v) sucrose, 0.8% (w/v) agar (solid medium) and 50 µg/mL kanamycin (when antibiotic selection was necessary). Plates were placed vertically in the growth chamber to allow root growth.

RT-quantitative PCR (RT-qPCR) and histochemical GUS analysis upon wounding of *AtCuAOβ* were performed on 7-day-old WT and *AtCuAOβ-promoter::GFP-GUS* seedlings respectively, grown for 6 days in solid medium (supplemented with kanamycin for *AtCuAOβ-promoter::GFP-GUS* seedlings) and then transferred to ½ MS salt mixture (pH 5.7) supplemented with 0.5 (w/v) sucrose (liquid medium) for one more day, as acclimation. Cotyledons from acclimated seedlings were cut with scissors soon after liquid medium exchange, and then incubated in a growth chamber prior to be sampled at 0, 1, 3, 6 and 24 h for RT-qPCR or incubated with GUS staining after 3 h from the injury. For RT-qPCR analysis, the samples were frozen in liquid nitrogen and then kept at −80 °C until RNA extraction was done.

Analysis under Laser Scanning Confocal Microscope (LSCM) of root protoxylem position and meristem size by propidium iodide (PI) staining as well as H_2_O_2_ accumulation by Amplex Ultra Red (AUR) staining were carried out on WT and *Atcuaoβ* mutant seedlings as previously described [10]. In brief, 7-day-old seedlings were selected for homogeneity in root length and then transferred onto fresh medium with or without 100 μM *N,N^1^*-dimethylthiourea (DMTU) or 100 µM putrescine (Put). After the transfer, seedlings were injured by cutting the cotyledonary leaf with scissors and after 6 h (AUR staining) or 3 days (PI staining) unwounded control and leaf-wounded plants were collected for analysis under LSCM of AUR and PI staining, respectively. The effect of leaf wounding on root growth was evaluated as the difference between the length measured at the onset of the wounding and that measured after 3 days, accordingly to previously data reported [10].

### 4.2. Genomic RNA Extraction, RT-PCR and RT-Quantitative PCR (RT-qPCR) Analysis

Total RNA was isolated from WT seedlings (100 mg) by using TRIzol^®^ Reagent (Invitrogen) following the manufacture’s instruction with slightly modifications. To eliminate traces of genomic DNA, RNA samples were treated with RNase-Free DNase Set (QIAGEN). Quantitative expression profiles of *AtCuAOβ* were determined by reverse transcription-quantitative polymerase chain reaction (RT-qPCR) on 7-day-old whole seedlings. In detail, RT-qPCR analysis was performed on DNase-treated RNA (4 µg) as follows. cDNA synthesis and PCR amplification were carried out using *GoTaq^®^ 2-Step RT-qPCR System200* (Promega) following manufacturer’s protocol. The first cDNA strand was synthesized using random and oligo *dT* primers in an *iCycler ^TM^ Thermal Cycler* (Bio-Rad) with the following parameters: 25 °C for 5 min, 42 °C for 60 min and 70 °C for 15 min. The PCRs were run in a Corbett RG6000 (Corbett Life Science, QIAGEN) utilizing the following program: 95 °C for 2 min then 40 cycles of 95 °C for 7 s and 60 °C for 40 s. The melting program ramps from 60 °C to 95 °C rising by 1 °C each step. *AtCuAOβ* specific primers were *RT-qPCR-AtCuAOβ-for*/*rev* [11]. Ubiquitin-conjugating enzyme 21 (*UBC21*, At5g25760) was used as reference gene (*UBC21-for/rev*; [43]). The software used to control the thermocycler and to analyze data was the Corbett Rotor-Gene 6000 Application Software (version 1.7, Build 87; Corbett Life Science, QIAGEN, Milan, Italy). Fold change in the expression of the *AtCuAOβ* gene were calculated according to the ΔΔC_q_ method as previously described [43,44].

### 4.3. Histochemical GUS Assay

Investigation of constitutive and inducible tissue-specific expressions was carried out by histochemical GUS analysis under light microscopy (LM). GUS staining was performed as previously described [45]. Samples were gently soaked in 90% (v/v) cold acetone for 30 min at −20 °C for prefixation and rinsed three times with 50 mM sodium phosphate buffer pH 7.0. After that, plant material was immersed in the staining solution [1 mM 5-bromo-4-chloro-3-indolyl-β-D-glucuronide, 2.5 mM potassium ferrocyanide, 2.5 mM potassium ferricyanide, 0.1% (v/v) Triton X-100, 10 mM EDTA, 50 mM sodium phosphate buffer, pH 7.0] under *vacuum*. For constitutive tissue-specific gene expression, the staining reaction proceeded overnight at 37 °C in dark. For tissue-specific wounding-inducible expression, the staining reaction was allowed to proceed until differences in the intensity between wounded and unwounded plants were detected. Chlorophyll was extracted by washings with ethanol/acetic acid ratio 1:3 (v/v) for 30 min, then with ethanol/acetic acid ratio 1:1 (v/v) for 30 min and finally with 70% ethanol. Samples were stored in 70% ethanol at 4 °C, prior to be observed under LM. Images were acquired by a Leica DFC450C digital camera applied to a Zeiss Axioplan2 microscope. Shown images of roots were reconstructed aligning overlapping micrographs of the same seedling.

### 4.4. Protoxylem Position and Meristem Size Analysis under LSCM by Cell Wall PI Staining and Bright-Field Examination of Root Tissues

Root apices from 10-day-old WT and *Atcuaoβ* unwounded control and leaf-wounded seedlings treated or not with 100 µM DMTU or with 100 µM Put for the last 3 days, were incubated for 5/10 min in PI (10 μg/mL) to highlight cell wall and protoxylem [46] and then observed under LSCM using a 488 nm argon laser, with a 600–680 nm band-pass filter and a 40× oil immersion objective. The PI staining was allowed to proceed until protoxylem was completely highlighted. Roots were concurrently analyzed by bright-field microscopy, using the same laser beam as described above. To analyze protoxylem maturation, the distance from the root apical meristem of the first protoxylem cell with fully developed secondary wall thickenings was measured following the method previously described [11]. The length of the meristematic zone was determined by measuring the distance between the quiescent center and the first elongating cell in the cortex cell file as well as the number of cortical cells in the same distance [10,47,48,49]. Images were obtained by serial aligning of overlapping micrographs of the same root by Photoshop Software (Adobe, San Jose, CA, USA). Protoxylem position (defined by the position of the first protoxylem cell with fully developed secondary cell wall thickenings [10]) and meristem size were estimated exploiting the Leica Application Suite Advanced Fluorescence software.

### 4.5. Hydrogen Peroxide in Situ Detection by AUR Staining

To reveal the in situ extracellular H_2_O_2_ accumulation, the fluorogenic peroxidase substrate AUR (Molecular Probes, Invitrogen, Carlsbad, CA, USA) was exploited [50] and the fluorescence of the peroxidase reaction-product was detected under LSCM in root apices from 7-day-old WT and *Atcuaoβ* unwounded control and leaf-wounded seedlings 6 h after the injury as previously described [10]. For the measurement of the AUR fluorescence intensity, five rectangles of approximate 65 μm^2^ for each analyzed root were drawn over the protoxylem maturation zone and the sum of the pixels corresponding to the fluorescence present in each rectangle was measured exploiting the quantitative analysis of the LAS-AF software used to acquire the confocal images.

### 4.6. Statistics

For RT-qPCR analysis of three biological replicates each with three technical replicates were performed (*n* = 3). The analysis by GUS staining of constitutive and inducible tissue-specific gene expression was performed on a minimum of 10 plants from three independent experiments, utilizing the most representative transgenic line [11]. Images from single representative experiments are shown. The analyses under LSCM of protoxylem position, meristem size and H_2_O_2_ accumulation after PI and AUR staining as well as root growth analysis were performed on three independent experiments on a minimum of ten plants per treatment and per genotype (*n* = 30), yielding reproducible results. Images from single representative experiments are shown. Statistical tests of RT-qPCR, protoxylem position, meristem size and root growth were performed using GraphPad Prism (GraphPad Software) with One-way ANOVA analysis followed by Sidak’s multiple comparison tests. Statistical significance of differences was evaluated by *p*-values. *ns*, not significant *p*-values > 0.05; *, **, *** and **** *p*-values ≤ 0.05, 0.01, 0.001 and 0.0001, respectively. The average values of fluorescence intensity for unwounded control, leaf-wounded and/or Put-treated plants were obtained by analyzing five roots for treatment, and five rectangles of approximately 65 μm^2^ for each analyzed root (*n* = 25).

## 5. Conclusions

Wounding-induced long-distance signaling propagates information from the leaf to the root triggering an H_2_O_2_-mediated early root protoxylem differentiation [10]. Among enzymatic sources of H_2_O_2_ in the cell wall, amine oxidases have been involved in both wounding responses in leaf and early protoxylem differentiation in root [11,18,36,37]. Here, we provide evidence that Arabidopsis amine oxidase β (AtCuAOβ) is the source of the tissue-specific H_2_O_2_ production triggered by the long-distance leaf-to-root signaling that is induced by leaf-wounding and leads to early root protoxylem differentiation. Although the physiological role of this root phenotypic alteration still needs to be unraveled, it could be related to an improved functionality of this organ in water uptake during stress conditions.

## Supplementary Materials

The following are available online at https://www.mdpi.com/2223-7747/9/2/249/s1, Figure S1: AUR fluorescence intensities of WT and mutant seedlings (*Atcuaoβ.1* and *Atcuaoβ.2*), Figure S2: In situ H_2_O_2_ detection by LSCM analysis, after AUR staining, of roots from 7-day-old control (unwounded and untreated), unwounded control and leaf-wounded (*Atcuaoβ.1* and *Atcuaoβ.2*) seedlings treated with Put, Table S1: Analysis of differences in root growth in leaf-wounded 10-days-old WT and *Atcuaoβ* mutant (*Atcuaoβ.1* and *Atcuaoβ.2*) seedlings grown in medium with or without DMTU and with or without Put, Table S2. Analysis of differences in meristem size in leaf-wounded 10-days-old WT and *Atcuaoβ* mutant (*Atcuaoβ.1* and *Atcuaoβ.2*) seedlings grown in medium with or without DMTU and with or without Put.

## Abbreviations

AOs	Amine oxidases
AtCuAOs	*Arabidopsis thaliana* copper containing amine oxidases
AUR	Amplex Ultra Red
GUS	β-glucuronidase
CuAOs	Copper containing amine oxidases
GFP	Green fluorescent protein
H_2_O_2_	Hydrogen peroxide
LSCM	Laser Scanning Confocal Microscope
LW	Leaf-wounded
MeJA	Methyl jasmonate
DMTU	*N,N^1^*-dimethylthiourea
PAs	Polyamines
PAOs	Flavin adenine dinucleotide depending polyamine oxidases/polyamine oxidases
PI	Propidium iodide
PCD	Programmed cell death
Put	Putrescine
RT-qPCR	Reverse transcription quantitative PCR
ROS	Reactive oxygen species
WT	Wild Type

## References

[1] Machado R.A.R., Arce C.C.M., McClure M.A., Baldwin I.T., Erb M. (2018). Aboveground herbivory induced jasmonates disproportionately reduce plant reproductive potential by facilitating root nematode infestation. Plant Cell Environ..

[2] Bezemer T.M., van Dam N.M. (2005). Linking aboveground and belowground interactions via induced plant defenses. Trends Ecol. Evol..

[3] Koo A.J., Gao X., Jones A.D., Howe G.A. (2009). A rapid wound signal activates the systemic synthesis of bioactive jasmonates in Arabidopsis. Plant J..

[4] Glauser G., Grata E., Dubugnon L., Rudaz S., Farmer E.E., Wolfender J.L. (2008). Spatial and temporal dynamics of jasmonate synthesis and accumulation in Arabidopsis in response to wounding. J. Biol. Chem..

[5] Mousavi S.A., Chauvin A., Pascaud F., Kellenberger S., Farmer E.E. (2013). GLUTAMATE RECEPTOR-LIKE genes mediate leaf-to-leaf wound signalling. Nature.

[6] Wang G., Hu C., Zhou J., Liu Y., Cai J., Pan C., Wang Y., Wu X., Shi K., Xia X. (2019). Systemic Root-Shoot Signaling Drives Jasmonate-Based Root Defense against Nematodes. Curr. Biol..

[7] Tretner C., Huth U., Hause B. (2008). Mechanostimulation of Medicago truncatula leads to enhanced levels of jasmonic acid. J. Exp. Bot..

[8] Hasegawa S., Sogabe Y., Asano T., Nakagawa T., Nakamura H., Kodama H., Ohta H., Yamaguchi K., Mueller M.J., Nishiuchi T. (2011). Gene expression analysis of wounding-induced root-to-shoot communication in Arabidopsis thaliana. Plant Cell Environ..

[9] Li C., Liu G., Xu C., Lee G.I., Bauer P., Ling H.Q., Ganal M.W., Howe G.A. (2003). The tomato suppressor of prosystemin-mediated responses2 gene encodes a fatty acid desaturase required for the biosynthesis of jasmonic acid and the production of a systemic wound signal for defense gene expression. Plant Cell.

[10] Fraudentali I., Rodrigues-Pousada R.A., Volpini A., Tavladoraki P., Angelini R., Cona A. (2018). Stress-Triggered Long-Distance Communication Leads to Phenotypic Plasticity: The Case of the Early Root Protoxylem Maturation Induced by Leaf Wounding in Arabidopsis. Plants.

[11] Ghuge S.A., Carucci A., Rodrigues Pousada R.A., Tisi A., Franchi S., Tavladoraki P., Angelini R., Cona A. (2015). The apoplastic copper AMINE OXIDASE1 mediates jasmonic acid-induced protoxylem differentiation in Arabidopsis roots. Plant Physiol..

[12] Topp C.N. (2016). Hope in Change: The Role of Root Plasticity in Crop Yield Stability. Plant Physiol..

[13] Prince S., Murphy M., Mutava R.N., Durnell L.A., Valliyodan B., Shannon J.G., Nguyen H.T. (2017). Root xylem plasticity to improve water use and yield in water-stressed soybean. J. Exp. Bot..

[14] Reymond P., Weber H., Damond M., Farmer E.E. (2000). Differential gene expression in response to mechanical wounding and insect feeding in Arabidopsis. Plant Cell.

[15] Creelman R.A., Mullet J.E. (1997). Biosynthesis and action of jasmonates in plants. Annu. Rev. Plant Physiol. Plant Mol. Biol..

[16] Ghuge S.A., Tisi A., Carucci A., Rodrigues-Pousada R.A., Franchi S., Tavladoraki P., Angelini R., Cona A. (2015). Cell wall amine oxidases: New players in root xylem differentiation under stress conditions. Plants.

[17] Tisi A., Federico R., Moreno S., Lucretti S., Moschou P.N., Roubelakis-Angelakis K.A., Angelini R., Cona A. (2011). Perturbation of polyamine catabolism can strongly affect root development and xylem differentiation. Plant Physiol..

[18] Cona A., Tisi A., Ghuge S.A., Franchi S., de Lorenzo G., Angelini R. (2014). Wound healing response and xylem differentiation in tobacco plants over-expressing a fungal endopolygalacturonase is mediated by copper amine oxidase activity. Plant Physiol. Biochem..

[19] Møller S.G., McPherson M.J. (1998). Developmental expression and biochemical analysis of the Arabidopsis atao1 gene encoding an H_2_O_2_-generating diamine oxidase. Plant J..

[20] Ghuge S.A., Carucci A., Rodrigues-Pousada R.A., Tisi A., Franchi S., Tavladoraki P., Angelini R., Cona A. (2015). The MeJA-inducible copper amine oxidase AtAO1 is expressed in xylem tissue and guard cells. Plant Signal. Behav..

[21] Tavladoraki P., Cona A., Angelini R. (2016). Copper-containing amine oxidases and FAD-dependent polyamine oxidases are key players in plant tissue differentiation and organ development. Front Plant Sci..

[22] Tavladoraki P., Cona A., Federico R., Tempera G., Viceconte N., Saccoccio S., Battaglia V., Toniello A., Agostinelli E. (2012). Polyamine catabolism: Target for antiproliferative therapies in animals and stress tolerance strategies in plants. Amino Acids.

[23] Moschou P.N., Wu J., Cona A., Tavladoraki P., Angelini R., Roubelakis-Angelakis K.A. (2012). The polyamines and their catabolic products are significant players in the turnover of nitrogenous molecules in plants. J. Exp. Bot..

[24] Rea G., de Pinto M.C., Tavazza R., Biondi S., Gobbi V., Ferrante P., de Gara L., Federico R., Angelini R., Tavladoraki P. (2004). Ectopic expression of maize polyamine oxidase and pea copper amine oxidase in the cell wall of tobacco plants. Plant Physiol..

[25] Moschou P.N., Paschalidis K.A., Delis I.D., Andriopoulou A.H., Lagiotis G.D., Yakoumakis D.I., Roubelakis-Angelakis K.A. (2008). Spermidine exodus and oxidation in the apoplast induced by abiotic stress is responsible for H2O2 signatures that direct tolerance responses in tobacco. Plant Cell.

[26] Marina M., Maiale S.J., Rossi F.R., Romero M.F., Rivas E.I., Gárriz A., Ruiz O.A., Pieckenstain F.L. (2008). Apoplastic polyamine oxidation plays different roles in local responses of tobacco to infection by the necrotrophic fungus Sclerotinia sclerotiorum and the biotrophic bacterium Pseudomonas viridiflava. Plant Physiol..

[27] Rodríguez A.A., Maiale S.J., Menéndez A.B., Ruiz O.A. (2009). Polyamine oxidase activity contributes to sustain maize leaf elongation under saline stress. J. Exp. Bot..

[28] Yamakawa H., Kamada H., Satoh M., Ohashi Y. (1998). Spermine is a salicylate-independent endogenous inducer for both tobacco acidic pathogenesis-related proteins and resistance against tobacco mosaic virus infection. Plant Physiol..

[29] Yoda H., Fujimura K., Takahashi H., Munemura I., Uchimiya H., Sano H. (2009). Polyamines as a common source of hydrogen peroxide in host- and nonhost hypersensitive response during pathogen infection. Plant Mol. Biol..

[30] Cona A., Cenci F., Cervelli M., Federico R., Mariottini P., Moreno S., Angelini R. (2003). Polyamine oxidase, a hydrogen peroxide-producing enzyme, is up-regulated by light and down-regulated by auxin in the outer tissues of the maize mesocotyl. Plant Physiol..

[31] Cona A., Moreno S., Cenci F., Federico R., Angelini R. (2005). Cellular redistribution of flavin-containing polyamine oxidase in differentiating root and mesocotyl of Zea mays L. seedlings. Planta.

[32] Schmidt R., Kunkowska A.B., Schippers J.H. (2016). Role of reactive oxygen species during cell expansion in leaves. Plant Physiol..

[33] Kimura S., Waszczak C., Hunter K., Wrzaczek M. (2017). Bound by Fate: The Role of Reactive Oxygen Species in Receptor-Like Kinase Signaling. Plant Cell.

[34] Cona A., Rea G., Angelini R., Federico R., Tavladoraki P. (2006). Functions of amine oxidases in plant development and defence. Trends Plant Sci..

[35] Kärkönen A., Kuchitsu K. (2015). Reactive oxygen species in cell wall metabolism and development in plants. Phytochemistry.

[36] Angelini R., Tisi A., Rea G., Chen M.M., Botta M., Federico R., Cona A. (2008). Involvement of polyamine oxidase in wound healing. Plant Physiol..

[37] Rea G.M., Metoui O., Infantino A., Federico R., Angelini R. (2002). Copper amine oxidase expression in defense responses to wounding and Ascochyta rabiei invasion. Plant Physiol..

[38] Duan Y., Zhang W., Li B., Wang Y., Li K., Sodmergen Han C., Zhang Y., Li X. (2010). An endoplasmic reticulum response pathway mediates programmed cell death of root tip induced by water stress in Arabidopsis. New Phytol..

[39] Liu Y., Xiong Y., Bassham D.C. (2009). Autophagy is required for tolerance of drought and salt stress in plants. Autophagy.

[40] Cui X., Ge C., Wang R., Wang H., Chen W., Fu Z., Jiang X., Li J., Wang Y. (2010). The BUD2 mutation affects plant architecture through altering cytokinin and auxin responses in Arabidopsis. Cell Res..

[41] Baima S., Forte V., Possenti M., Peñalosa A., Leoni G., Salvi S., Felici B., Ruberti I., Morelli G. (2014). Negative feedback regulation of auxin signaling by ATHB8/ACL5 BUD2 transcription module. Mol. Plant..

[42] Valvekens D., Montagu M.V., Van Lijsebettens M. (1988). Agrobacterium tumefaciens-mediated transformation of Arabidopsis thaliana root explants by using kanamycin selection. Proc. Natl. Acad. Sci. USA.

[43] Fraudentali I., Ghuge S.A., Carucci A., Tavladoraki P., Angelini R., Cona A., Rodrigues-Pousada R.A. (2019). The copper amine oxidase AtCuAOδ participates in abscisic acid-induced stomatal closure in Arabidopsis. Plants.

[44] Livak K.J., Schmittgen T.D. (2001). Analysis of relative gene expression data using real-time quantitative PCR and the 2 (Delta Delta C(T)) Method. Methods.

[45] Jefferson R.A. (1987). Assaying chimeric genes in plants: The GUS gene fusion system. Plant Mol. Biol..

[46] Mähönen A.P., ten Tusscher K., Siligato R., Smetana O., Díaz-Triviño S., Salojärvi J., Wachsman G., Prasad K., Heidstra R., Scheres B. (2014). PLETHORA gradient formation mechanism separates auxin responses. Nature.

[47] Chen Q., Sun J., Zhai Q., Zhou W., Qi L., Xu L., Wang B., Chen R., Jiang H., Qi J. (2011). The basic helix-loop-helix transcription factor MYC2 directly represses PLETHORA expression during jasmonate-mediated modulation of the root stem cell niche in Arabidopsis. Plant Cell.

[48] Casamitjana-Martínez E., Hofhuis H.F., Xu J., Liu C.M., Heidstra R., Scheres B. (2003). Root-specific CLE19 overexpression and the sol1/2 suppressors implicate a CLV-like pathway in the control of Arabidopsis root meristem maintenance. Curr. Biol..

[49] Dello Ioio R., Nakamura K., Moubayidin L., Perilli S., Taniguchi M., Morita M.T., Aoyama T., Costantino P., Sabatini S. (2008). A genetic framework for the control of cell division and differentiation in the root meristem. Science.

[50] Ashtamker C., Kiss V., Sagi M., Davydov O., Fluhr R. (2007). Diverse subcellular locations of cryptogein-induced reactive oxygen species production in tobacco Bright Yellow-2 cells. Plant Physiol..

