# Supplementary material for: Leaf-Wounding Long-Distance Signaling Targets AtCuAOβ Leading to Root Phenotypic Plasticity
Source: Plants (Basel). 2020 Feb 15;9(2):249. doi: 10.3390/plants9020249 (PMC7076439; doi:10.3390/plants9020249)
Supplement: Supplementary file 1 [file plants-09-00249-s001.pdf]

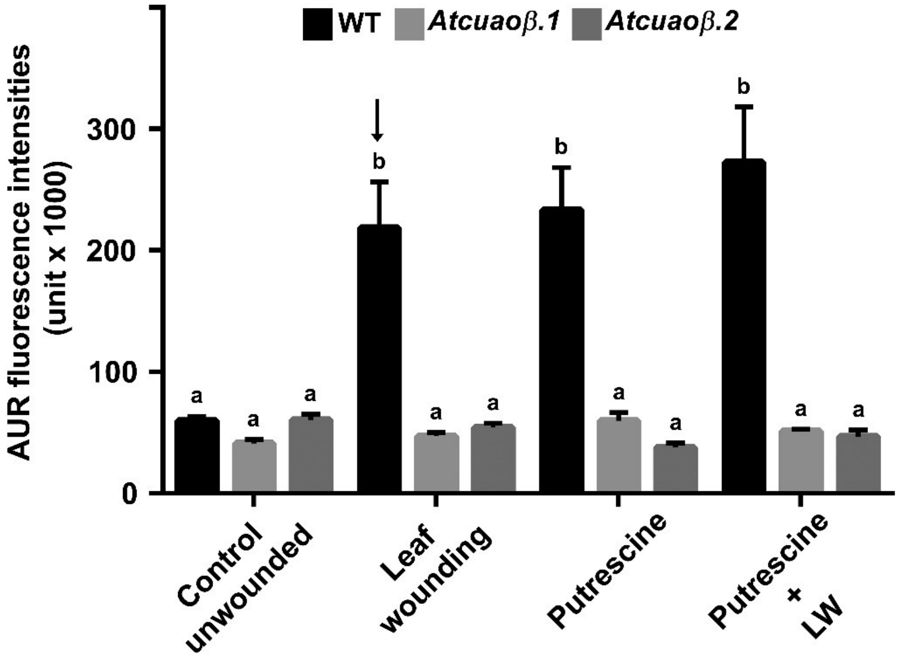

Figure S1. H_2O_2-dependent AUR fluorescence intensities in unwounded untreated control, Put-treated and leaf-wounded Put-treated WT, *Atcuaoβ.1* and *Atcuaoβ.2* mutant seedlings, measured as the sum of the pixels of each of five 65 μm^2 rectangles for each condition (mean values ± SD; $n = 25$). The statistical significance levels (*P*-values) were evaluated with one-way analysis of variance (ANOVA) followed by Sidak's multiple comparison test levels. *P*-values of similar significance are indicated with letters.

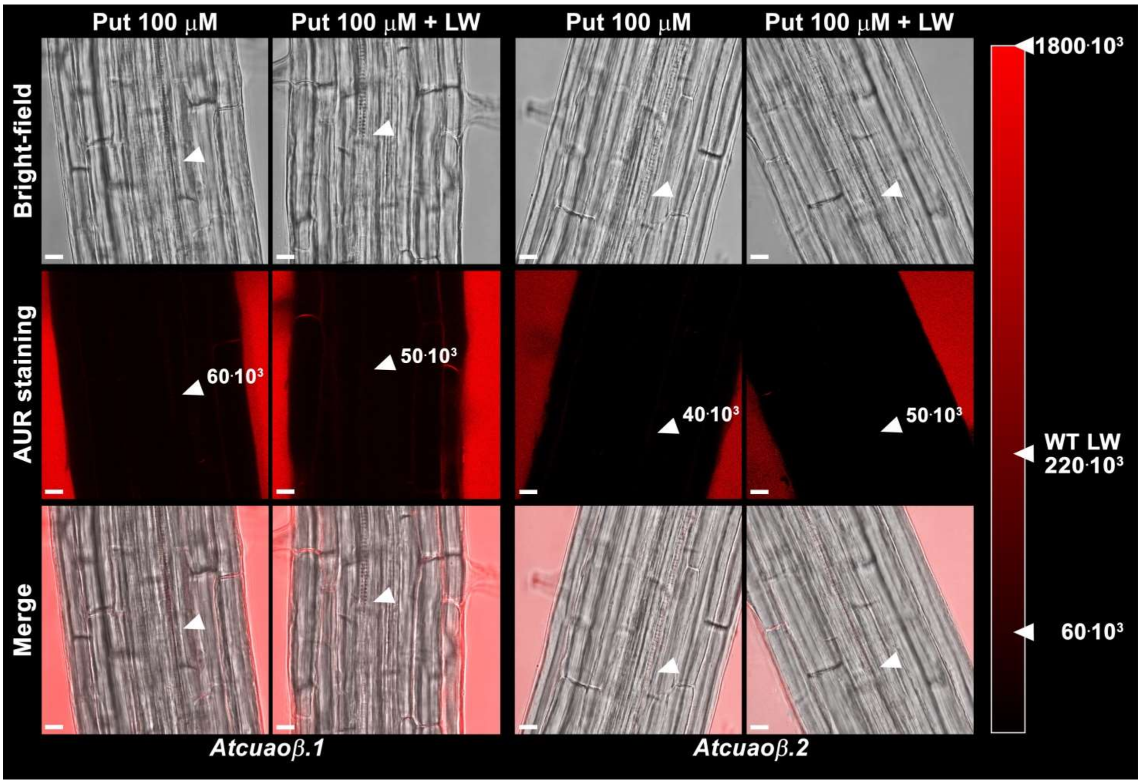

Figure S2. *In situ* H_2O_2 detection by LSCM analysis, after AUR staining, of roots from 7-day-old unwounded Put-treated (Put 100 μM) and leaf-wounded Put-treated (Put 100 μM+ LW) *Atcuaoβ.1* and *Atcuaoβ.2* seedlings. Treatment with 100 μM Put was performed 6 hours after the injury. The corresponding bright-field and overlay images are shown. Micrographs show the root zone corresponding to the site of appearance of the first protoxylem cell with fully developed secondary cell wall thickenings (arrows) and have been taken at the level of the central root section. Images are representative of those obtained from ten seedlings from three independent experiments. The average values of fluorescence intensity measured as the sum of the pixels of each of five 65 μm^2 rectangle are reported for each condition (mean values ± SD; n = 25). The maximum pixel sum for a completely saturated square was approximately 1800×10^3. In the red degrading scale are reported for comparison the average values of fluorescence intensity for unwounded and leaf-wounded (Put-untreated) WT plants that were, respectively, $60\times10^3 \pm 19\times10^3$ and $220\times10^3 \pm 38\times10^3$ (data from Fraudentali *et al*., 2018 [10]). Bars: 10 μm.

Table S1. Analysis of differences in root growth in leaf-wounded 10-days-old WT and *Atcuaoβ* mutant (*Atcuaoβ.1* and *Atcuaoβ.2*) seedlings grown in medium with or without 100 μM DMTU and with or without 100 μM Put. The effect of leaf wounding/treatments on root growth was evaluated as the difference between the length measured at the onset of the wounding/treatment and that measured after 3 days. These experiments were repeated at least three times with ten seedlings analyzed each time (mean values ± SD; $n = 30$). The statistical significance levels (*P*-values) were evaluated with one-way analysis of variance (ANOVA) followed by Sidak's multiple comparison test levels. *P*-values between unwounded/untreated control and wounded/treated plants were not significant.

	**Root Growth (cm)**		
	**Wild-type**	***Atcuaoβ.1***	***Atcuaoβ.2***
Control	2.34 ± 0.23	2.34 ± 0.22 ns	2.33 ± 0.21 ns
Leaf wounding	2.26 ± 0.35	2.38 ± 0.25 ns	2.22 ± 0.25 ns
DMTU 100 μM	2.31 ± 0.22	2.35 ± 0.26 ns	2.32 ± 0.34 ns
Leaf Wounding + DMTU 100 μM	2.37 ± 0.22	2.31 ± 0.28 ns	2.24 ± 0.25 ns
Put 100 μM	2.32 ± 0.25	2.24 ± 0.35 ns	2.35 ± 0.28 ns
Leaf Wounding + Put 100 μM	2.31 ± 0.26	2.31 ± 0.21 ns	2.37 ± 0.28 ns

Table S2. Analysis of differences in meristem size in leaf-wounded 10-days-old WT and *Atcuaoβ* mutant (*Atcuaoβ.1* and *Atcuaoβ.2*) seedlings grown in medium with or without 100 μM DMTU and with or without 100 μM Put. The length of the meristematic zone was determined by measuring the distance between the quiescent center and the first elongating cell in the cortex cell file. In the case of WT and *Atcuaoβ.1* the number of cortical cells corresponding to the measured length was counted. These experiments were repeated at least three times with ten seedlings analyzed each time (mean values ± SD; $n = 30$). The statistical significance levels (*P*-values) were evaluated with one-way analysis of variance (ANOVA) followed by Sidak's multiple comparison test levels. *P*-values between unwounded/untreated control and wounded/treated plants were not significant.

	**Meristem Size (μm)**		
	**Wild-type**	***Atcuaoβ.1***	***Atcuaoβ.2***
Control	350,7 ± 16,7	339,9 ± 14,4	355,7 ± 8,4
Leaf wounding	335,6 ± 13,4	331,7 ± 16,0	345,0 ± 20,9
DMTU 100 μM	353,4 ± 23,7	341,4 ± 7,5	350,8 ± 11,6
Leaf Wounding + DMTU 100 μM	346,8 ± 28,2	345,0 ± 17,0	341,3 ± 17,5
Put 100 μM	337,3 ± 17,5	336,1 ± 17,1	345,0 ± 10,0
Leaf Wounding + Put 100 μM	351,6 ± 35,3	338,3 ± 12,5	350,0 ± 10,8

	**Meristem Size (n.° cortical cells)**	
	**Wild-type**	***Atcuaoβ.1***
Control	44,0 ± 6,1	41,6 ± 5,1
Leaf wounding	48,0± 4,2	45,0 ± 3,7
Put 100 μM	44,3± 1,5	41,0 ± 6,2
Leaf Wounding + Put 100 μM	45,0± 2,0	42,7 ± 3,5
